# Recent Patents on Nasal Vaccines Containing Nanoadjuvants

**DOI:** 10.2174/2667387816666220420124648

**Published:** 2022-10-19

**Authors:** Francesco Candela, Eride Quarta, Francesca Buttini, Adolfo Ancona, Ruggero Bettini, Fabio Sonvico

**Affiliations:** 1 Department of Food and Drug, University of Parma, Parco Area delle Scienze 27/A, 43124 Parma, Italy;; 2 University Centre for Innovation in Health Products (Biopharmanet-TEC), University of Parma, Parco Area delle Scienze 27/A, 43124 Parma, Italy

**Keywords:** Nanoadjuvants, nanoemulsion, nanoparticles, nanotechnology, nasal vaccine, cellular immunity

## Abstract

Vaccines are one of the greatest medical achievements of modern medicine. The nasal mucosa represents an effective route of vaccination for both mucosal immunity and peripheral, being at the same time an inductive and effector site of immunity. In this paper, the innovative and patented compositions and manufacturing procedures of nanomaterials have been studied using the peer-reviewed research literature. Nanomaterials have several properties that make them unique as adjuvant for vaccines. Nanoadjuvants through the influence of antigen availability over time affect the immune response. Namely, the amount of antigen reaching the immune system or its release over prolonged periods of time can be effectively increased by nanoadjuvants. Mucosal vaccines are an interesting alternative for immunization of diseases in which pathogens access the body through these epithelia. Nanometric adjuvants are not only a viable approach to improve the efficacy of nasal vaccines but in most of the cases they represent the core of the intellectual property related to the innovative vaccine.

## 1 INTRODUCTION

The continuous emergence of new pathogens and the evolution of resistance strains toward drugs in clinical use make the main goal of modern medical science, that is the development of innovative and efficient vaccination strategies capable of generating long-lasting effective immunity against infections. The current vaccine market is also expected to expand as a consequence of the use of novel and alternative routes of vaccine administration. Traditionally, most vaccines are administered parenterally, but nowadays alternative routes of vaccination, particularly mucosal administration, are increasingly being explored and are the subject of extensive research [1]. The nasal cavity offers unique opportunities for convenient administration of vaccines to prevent infectious diseases compared with parental administration, not only because it offers better compliance due to ease of administration, but also because it has permeable epithelium and high availability of immunoreactive sites. Nasal cavities are an anatomically attractive route for immunisation: the mucosal surface is easily accessible, highly vascularised, and offers a relatively large absorption surface, generated by the large number of microvilli covering the nasal epithelial cells. This approach is interesting, especially for the prevention of those infectious.Diseases transmitted by pathogens entering the body through the mucosal surfaces of the airways. Other advantages of nasal immunization are that it can induce both antigen-specific antibody production and a cellular memory response in the respiratory tract as well as in other mucosal tissues [2]. Indeed, a vaccine may be more efficient if it can elicit both humoral and cellular immune responses. However, conventional vaccines can often induce only strong antibody responses but limited cellular immunity [3]. The advantages of mucosal routes of vaccine administration, such as ease of self-administration and non-invasiveness, could lead to an overall improvement in patient compliance, especially in paediatric patients. In addition, the administration of mucosal vaccines does not necessarily require trained personnel, which reduces costs and facilitates their use in mass vaccination programs. Indeed, the nose appears to be an attractive route for rapid immunization of large populations, as it does not require needles and syringes, which are a potential source of infection. In the formulation of nasal vaccines (but this is true for innovative vaccines in general) adjuvants are key components for the effectiveness of the vaccine. In fact, adjuvants are vaccine components capable of enhancing and/or directing antigen-specific immune responses. These effects are often sought for vaccines based on rationally designed recombinant antigens that present excellent safety, but lower immunogenicity compared with vaccines based on live-attenuated or inactivated pathogens [4]. In this sense, the application of pharmaceutical nanotechnologies has emerged as one of the most interesting approaches to design particulate systems able to efficiently encapsulate and protect antigens, target the mucosalimmune system and incorporate and/or act themselves as mucosal adjuvants maximizing immune response [5].

In the last decade, several reviews have been published concerning mucosal vaccination, specifically highlighting the nasal route as a promising strategy for drug and vaccine delivery. Mato *et al.* highlighted the advantages and characteristics of the nasal route for vaccine delivery [6]. The nanotechnology applications for different administration routes, among them the intranasal delivery and the discussion of the factors affecting the regulatory aspects and patient expectation of this delivery was the focus of the review by Alshweiat *et al*. [7]. Also, the state of the art of nanomaterial employed for nasal nanovaccines in animals and humans has been reviewed [8]. In this work the technological aspect of innovative formulations and manufacturing procedures of nanomaterials proposed as adjuvants, have been studied based on patents and peer-reviewed literature. In particular, how the nanometric adjuvants affect the immune response represents the core of the intellectual property protected in the reviewed innovative vaccine strategies.

### 1.1 Nasal Cavity

The nose is divided into two large cavities, consisting of 14 bones connected by a tough fibrous membrane, forming a roof, floor, and inner and outer walls. The nasal passage is about 12-14 cm long and about 5 cm high, and the total surface area of both nasal cavities is 160 cm^2^, which increases to 96,000 cm^2^ when the microvillus of the nasal epithelium are taken into account; the total volume is about 15 ml [9]. Each cavity extends from the base of the skull to the roof of the mouth, opening to the face through the nostril (nares anterior) and extending to an oval opening in the upper part of the nasopharynx (pharynx). The nasal septum also called the internal wall separates the two nasal cavities. This wall is thickest at the upper edge and has deep furrows traversed by numerous vascular and nerve channels through which the nasopalatine nerve, among others, passes. The outer wall of the nasal cavities has three folds, the superior, middle, and internal turbinate (or conchae), which increase the resistance of the nasal passages, cause turbulence in the airflow, and promote contact between the air and the mucosal surface [9]. The nasal vestibule has the smallest cross-sectional area in the respiratory tract (about 0.3 cm^2^ on each side) and extends from the entrance of the nostrils. It is lined with skin containing hairs called vibrissae and sweat and sebaceous glands. The vibrissae guard the anterior end of the inferior turbinate (conchae inferior). The nasal cavity is permeated with arteries, veins, lymphatic vessels, and neurons. The nasal mucosa is highly vascularised with a network of cavernous or fenestrated veins beneath the mucosa. The fenestrate always face the respiratory epithelium and are thought to be one of the sources of fluid for humidification [9].

The nasal mucosa consists of seven cell types, four of which are in the respiratory region and five in the olfactory region. Their density and distribution vary greatly from one region of the nose to another [10, 11]. The cells of the nasal epithelium are:

• Ciliated cells: these are the most common cell in the nasal epithelium and their motility is pivotal for ciliary clearance. This is a protective mechanism that eliminates pathogens and pollutants present, trapping them and removing mucus where inhaled particles impact. Because of this clearance, inhaled drugs have only a short window of time to be absorbed, as they are eventually flushed from the pharynx and swallowed. Ciliated cells have a large number of mitochondria, which is a consequence of considerable metabolic activity. Ciliated cells are also covered with approximately 300 microvilli, which greatly increase the surface area available for absorption.

• Basal cells: these cells play an important role in replacing other cell types in the epithelium. Due to their pluripotency, they are able to differentiate and renew other epithelial cell types.

• Columnar cells: these are the cells without cilia that lie on the basement membrane and extend to the lumen of the airways, where they develop microvilli that increase their surface area 600-fold. Several pharmacologically active substances, including proteins, can be taken up by the columella cells. The influenza virus also employs this cell type to cross the nasal mucosa.

• Goblet cells: these cells are sometimes called mucus cells or chalice cells. They are distributed among other cells throughout the epithelium. These cells are mucin-secreting cells or unicellular glands, although their contribution to the volume of nasal secretion is probably small, compared with the submucosal glands (6).

• Olfactory cells: these cells are bipolar neurons placed in the nose olfactory region and the vomeronasal organ, and they are transducers of odorant interaction with chemoreceptors of neural signals.

• Supporting cells: the olfactory cells need support cells called sustentacular cells, which are the most numerous cells in the olfactory epithelium providing metabolic and physical support and protection to the olfactory cells.

• Brush cells: their function is that of receptor cells for general sensations like irritation or other forms of mucosasensory stimulation. They have large microvilli on their apical surface and the basal surface is in synaptic contact with the trigeminal nerve terminals that penetrate the basal membrane.

The nasal mucosa is the first point of contact with inhaled antigens; hence, it is an important part of the mucosal immune system and provides an efficient vaccine pathway for peripheral and mucosal immunity, being both an inductive and an effector site of immunity. The mucosal immune system can be shared into two general areas known as inductive and effector sites. Inductive sites are areas where antigen uptake leads to initial activation of immune cells, while effector sites allow antibodies and cells of the immune system to perform their specific function after activation [5, 12]. In addition, intranasal immunisation can elicit both local and systemic immune responses at mucosal sites and even at distant mucosal sites as effector immune cells spread throughout the general mucosal immune system. The upper and the lower airways have an immune system that can be divided into three parts: an epithelial compartment at the surface of the epithelium and the underlying connective tissue containing immunocompetent cells; the lymphoid structures of the nose and the bronchus-associated epithelium, *i.e*., nasal-associated lymphoid tissues (NALT), laryngeal-associated lymphoid tissues (LALT), bronchus-associated lymphoid tissues (BALT); lymph nodes, draining the respiratory system.

Various cell types of the immune system such as macrophages, dendritic cells, M-cells, and intraepithelial lymphocytes form the respiratory tract epithelial barrier. The latter cell type appears to be limited in the respiratory tract. Mucosal immune responses are thought to be triggered primarily by the MALT, which does not have a lymphatic supply of antigens. As a consequence, nasal vaccines can be designed for clinical use if the inductive conditions of this route of administration are activated, in particular the migratory properties of mucosal B-cells [13].

The mucosal surface protection against pathogens can be divided into two general defence systems: innate and adaptive immune defences. The physical and chemical action of the mucous membranes is fundamental to innate defense.

The epithelia of the mucosal surfaces of the respiratory tract (as well as other mucosal surfaces) and associated glands produce nonspecific or innate defenses, including mucins and antimicrobial proteins. Lysozyme, lactoferrin, and β-defensin are some of them. In addition, epithelial cells are active initiators of other mucosal defence mechanisms. Namely, they act as sensors that detect dangerous microbial components *via* pattern-recognition receptors such as Toll-like receptors (TLRs). They answer by sending cytokine and chemokine signals to be underlying mucosal cells, dendritic cells (DCs), and macrophages, to activate innate nonspecific defence mechanisms but also adaptive immune responses [14, 15].

Adaptive immune protection of mucosal surfaces is a defense system in which specialised immune cells produce and secrete dimeric or multimeric immunoglobulin A antibodies (sIgA) that are resistant to degradation [16]. IgAs are synthesized by lymphocytes B under cytokine stimulation (TGF-β and IL-5). The secretory IgA during their synthesis undergo a high degree of glycosylation and formation of dimeric or multimeric antibodies, which are the reason for the IgA protease resistance [17]. According to the mechanism of “immunity” promoted by sIgA, antigens and microorganisms are trapped in mucus, preventing direct contact of pathogens with the mucosal surface. Conversely, microbial surface molecules that mediate attack on the epithelium can be blocked or hindered by other specific IgAs [18]. Moreover, sIgAs could scavenge pathogens that enter the vesicular compartments of epithelial cells during polymer Ig receptor-mediated transcytosis (pIgR) or neutralize pathogen-derived toxins and adhesions [19]. In addition, sIgAs facilitate the uptake of pathogens into isolated lymphoid compartments and the presentation of antigens to dendritic cells (DCs). Recognition of sIgA by dendritic cells induces regulation of their secretion and production by inhibiting the secretion of IL -12 cytokines and leads to induction of T-helper 2 (Th2) or regulatory T-cell (Treg) responses [20]. Administration of vaccines to mucosal surfaces or antigens promotes the local synthesis of IgG. This mechanism can potentially neutralize pathogens that prevent systemic spread by penetrating mucosal surfaces. Another type of protection against infection is that specific cytotoxic T lymphocytes (CTL) or antibody-dependent cell-mediated cytotoxicity (ADCC), cooperative protection between natural killer (NK) cells and antibodies, do not prevent pathogen invasion but kill it, especially in viral infections [21].

The main aim of vaccination is to promote the development of a durable protective immune response. This can be obtained by the induction of specific T- and B-memory cells together with the presence of some readily available antibodies. The parenteral injection of attenuated non-pathogenic microorganisms allows to reach this objective, despite this approach entails the reversion microorganisms risk to a pathogenic state. Currently, another approach has been developed in which purified antigens and custom-made epitopes are used because of their intrinsic safety. However, these purified antigens are typically unable to build by themselves an adequate immune response. This lack of potency is another challenge in developing a vaccine for mucosal administration. To overcome this challenge, the use of an appropriate vaccine adjuvant is critical.

To perform the function of immunostimulants and/or immunomodulators, vaccine adjuvants are administered together with antigens. Therefore, they are of large interest in improving immune responses through innovative and safer vaccination strategies [22, 23]. Following nasal vaccination, adjuvants may modulate or enhance the immune response by several mechanisms. These include enhancement of antigen immune availability, recognition of “foreign/dangerous” signals, and immunomodulation [24].

Adjuvants such as micro- and nanoparticles, liposomes, and particulate vectors are vaccine carriers that enhance antigen immunogenicity by improving its immunoavailability [25]. Specialized antigen-presenting cells, of which DC are the most important, take up and process the antigen. Naïve DCs circulating in the mucosal tissue can take up antigens, which initiate their maturation. After maturation, DCs migrate to secondary lymphoid tissues where they can elicit a Th or CTL response. In order to perform this function, a sufficient amount of DCs must reach secondary lymphoid tissues by crossing several barriers (such as mucosal as well as enzyme degradation of the antigen) after nasal administration [24]. Incorporating an antigen into the matrix of a suitable carrier can improve the stability of the antigen in a biological environment [26]. Hence, the similarity of the antigen-loaded carrier to the pathogen/antigen disposition leads to higher immunogenicity of the antigen. For this reason, it has been proposed that surfaces with highly repetitive antigen sequences can cross-link B-cell receptors and thereby elicit efficient humoral immune responses [27, 28].

The immune system can recognize some of the molecular patterns characteristic of pathogens such as the “pathogen-associated molecular patterns” (PAMP), recognized by DCs through specialized receptors such as Toll-like receptors (TLR), mannose receptors and complement receptors [29, 30]. Some foreign/danger signals may be presented by adjuvants to enhance immune system co-stimulation signaling. Examples of adjuvants that play this role include: poly I:C, bacterial lipopolysaccharides, monophosphoryl lipid A, and mannose. Moreover, chitosan has been shown to interact with these receptors contributing to a co-stimulation of DCs [31-33].

Adaptive immune responses are enhanced by T-cells when exposed to antigen epitopes and some other costimulatory molecules. T-helper type 1 (Th1) lymphocytes secrete interferon-gamma (IFN-γ), which promotes the proliferation of macrophages and enhancement of the microbicidal activity of various cell populations, as well as other processes of cellular immunity. Helper T-lymphocytes type 2 (Th2) produce interleukin-4 (IL-4) and they stimulate humoral responses [24]. The Th1/Th2 ratio of immune responses can sometimes be altered by the antigens and adjuvants [34]. This differentiation has not yet been clearly demonstrated, but some work has shown that some adjuvants such as ISCOMs are involved in the Th1 response [35], whereas cholera toxins or a few polysaccharides tend to produce a Th2 response [36].

### 1.2 Nanoadjuvants

Nanoadjuvants are nanomaterials that play a key role in increasing the amount of antigen that reaches the immune system. In addition, nanoadjuvants are able to control the release of the antigen over longer periods of time. These properties of nanoadjuvants allow them to influence the availability of the antigen over a prolonged period of time, thereby affecting the immune response. The primary function of nanoadjuvants as antigen delivery systems can be combined with other effects, such as immunomodulation and/or immunostimulant [24, 37].

#### 1.2.1 Lipid-based Nanocarriers

The following section provides different typologies of lipid-based nanocarriers such as liposomes, ICOSMs, and other common lipid-based nanocarriers (multivesicular structures, virosomes, proteosomes, and proteoliposomes).

##### 1.2.1.1 Liposomes

Liposomes are structured as bilayered vesicles composed of lipids and cholesterol surrounding an aqueous core. As a result, they have both hydrophilic and hydrophobic structures that allow the uptake of a variety of antigens. Liposomes are preferentially endocytosed by macrophages [38]. Liposomes immunostimulatory properties are linked to their capacity to associate and release antigens over a prolonged time and to their preferential uptake by APCs that promote maturation and antigen preservation [39]. Parenteral administration of antigens in liposomes mostly induces preferential expression of IgG1, and therefore an immune system Th2 type of response that regulates the humoral response by activating B-lymphocytes. Albeit a Th1 response was also stated [40, 41]. Liposomes are interesting carriers for nasal administration of vaccines, which, thanks to their ability to cross the epithelial barrier, can enhance mucosal immunity to associated antigens when the system is administered intranasally [42]. Overall, systemic responses to antigens administered intranasally in liposomes were always significantly lower than those to antigens by parenteral administration [24]. Immunization of mucous membranes with liposomes appears to be limited and highly dependent on the concomitant administration of other co-stimulants, probably due to their rapid endocytosis by macrophages [43].

##### 1.2.1.2 Immunostimulatory Complexes or ISCOMs

Immunostimulatory Complexes or ISCOMs are self-assembled colloidal structures obtained from cholesterol, phospholipids a Quillaja saponin (Quil A^®^) and the antigen. ISCOMs have small sizes (around 40nm) and are a cable of targeting M-cells thanks to their multimeric presentation of antigen and the powerful immunostimulatory activity provided by saponin. Antigens bind to ISCOMs by electrostatic or hydrophobic interactions thanks to the negative surface area of ISCOMs due to the presence of glucuronic acid [44]. DCs readily take up ISCOMs, which are efficiently presented to T-lymphocytes [45]. After nasal inoculation, specific local and systemic immune responses are triggered by the antigens entrapped by ISCOMs [46].

ISCOMS cannot incorporate large and hydrophilic antigens. Due to its haemolytic activity and injection site reactions, the adjuvant Quil A has only been used in animal vaccines. However, when Quil A is incorporated into the ISCOM structure, these side effects appear to be almost absent [47, 48]. Therefore, the toxicity of Quil A is significantly lower when ISCOMs are administered nasally compared with parenteral administration [1]. ISCOMs preferentially induce Th1 and CTL responses, so recent work shows that ISCOMs could potentially be of interest for vaccination against intracellular parasites and for the treatment of some specific cancers by traditional parenteral administration rather than nasal administration, where other carriers appear to be more effective [49-53].

##### 1.2.1.3 Other Lipid-based Nanocarriers

In the context of transporters on the order of 1 µm, there are lipid-based nanocarriers that exert the function of adjuvants on antigenicity [24]. Mucosal responses (IgA and IgG) are stimulated by multivesicular structures (1-20 µm) containing lipoprotein A (from *Actinobacillus pleuropneumoniae*) and administered together with the immunostimulant CpG [54]. Recent studies have shown that the immunization regimen in mice treated with the DNA and antigen in combination with cationic lipid carriers enhanced the mucosal (IgA) and systemic (IgG and IgA) antibody responses related to the antigen without carriers. This new cationic lipid, designated N3, was developed in the form of an emulsion for DNA vaccine delivery. In another study, the same group used this N3 lipid as an adjuvant for intranasal administration of a DNA vaccine against HIV with multiple charges/multigene. The results confirmed that this vaccine could induce IgA responses in both rectal and vaginal mucosa, as well as systemic humoral (IgG) and cellular responses [55].

##### 1.2.1.4 Virosomes

Virosomes are produced by solubilizing glycoproteins extracted from viral particles and phospholipids and then removing the solubilising surfactant, thereby mimicking viral structures [56, 57]. The viral surface glycoproteins have a high affinity for mucosal surfaces, and the viral component in virosomes increases their efficiency in APC binding, allowing efficient induction of Th1 and Th2 immune responses as well as strong CTL responses [58, 59]. Virosomes are remarkable nasal carriers for various antigens, for example in DNA vaccines [60].

##### 1.2.1.5 Proteosomes

Proteosomes are vesicles that originated from the bacterial outer membrane. They are prepared by solubilising the outer membranes of bacteria, followed by ammonium sulphate precipitation and dialysis against a buffer containing detergent. Proteasomes, which are composed of proteins, are considered mavericks among lipid carriers [61]. Proteasome vaccine particles, which have hydrophobic moieties, can form a hydrophobic complex with antigens [1]. Proteoliposomes also belong to the same vector family. They are nanovesicles containing important outer membrane proteins and LPS of specific pathogens. When administered nasally, they elicit a systemic immune response and a higher than average local IgA response. However, the proteoliposome stability in an aqueous solution is an issue yet to be overcome [1, 62].

##### 1.2.1.6 Nanoemulsions

**Fig. (1):**
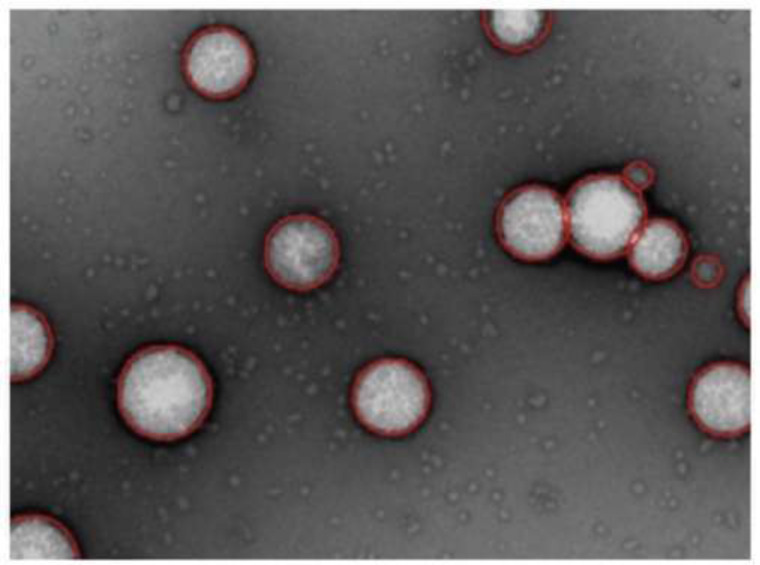
TEM image of silicate particle-stabilized oil in water nanoemulsion [64].

Nanoemulsions are drug delivery systems constituted of water systems and emulsified oil with average droplet diameters ranging from 50 to 1000 nm. Typically, the average droplet size of nanoemulsions ranges from 100 to 500 nm and they can exist as oil-in-water (o/w) or water-in-oil (w/o) forms, with the core of the particle being either oil or water, respectively. Nanoemulsions claim to dissolve large amounts of poorly soluble drugs and protect the drugs from hydrolysis and enzymatic degradation (Fig. **1**) [63-65].

The preparation of nanoemulsions as a nonequilibrium system of structured liquids requires a high-energy input or the use of surfactants, or both. A surfactant keeps the surface tension slow between oil and water. Small molecules, such as small nonionic surfactants, lower surface tension better than polymeric surfactants, such as polyvinyl alcohol. During emulsification, an emulsifier can also avoid shear-induced coalescence. The prerequisite for this is that the surfactant is present in the continuous phase in significant excess. This excess prevents shear-induced coalescence by allowing a new surface of the formed nanoscale droplets to be rapidly coated during the process. The excess surfactant typically forms micelles in the continuous phase that dissociate into monomers that can adsorb onto the surfaces of the newly formed droplets. Nanoemulsion formulations cost more energy than producing a macroemulsion.

There are various techniques and methods for preparing nanoemulsions, which can be divided into high-energy and low-energy emulsification methods. High energy manufacturing methods use mechanical devices that generate strong disruptive forces that break up the oil and water phases to form nano-sized droplets. This can be achieved with ultrasonic devices, microfluidizers and high-pressure homogenizers. High-pressure homogenization is a technique that produces small particle sizes (down to 1 nm) using a piston-driven high-pressure homogenizer. Microfluidization is a patented mixing technique that uses a microfluidizer, a device that uses a high-pressure positive displacement pump to force product through microchannels. Time, temperature, and mechanical properties of the device were used to affect the particle size of the dispersed phase droplets. This type of preparation may not be suitable for heat-sensitive drugs such as retinoids, and macromolecules such as proteins, enzymes and nucleic acids.

Nanoemulsions can also be prepared using a low-energy emulsification method that can promote the formation of ultra small droplets. Some low-energy techniques that may be applicable include self-emulsification, phase transition, and phase inversion temperature methods. These methods use the “stored” energy of the system to form small droplets. Changing parameters that affect the hydrophilic-lipophilic balance (HLB) of the system (temperature, composition, *etc*.), the process can be nonspontaneous, so energy can be usually required. *Phase inversion* is a temperature-dependent technique that uses the temperature-dependent solubility of nonionic surfactants, like polyethoxylated surfactants, to change their affinities for water and oil as a function of temperature, leading to the formation of nanoemulsions by slowing down the external phase. The Solvent Displacement Method is a method for the spontaneous construction of nanoemulsions, in which the oily phase is dissolved in water-miscible organic solvents, such as ethanol, acetone and ethyl methyl ketone finally added to an aqueous phase. Evaporation under vacuum is a technique suitable to remove organic solvent from the nanoemulsion.

##### 1.2.1.7 Self-Nanoemulsification Method

**Fig. (2):**
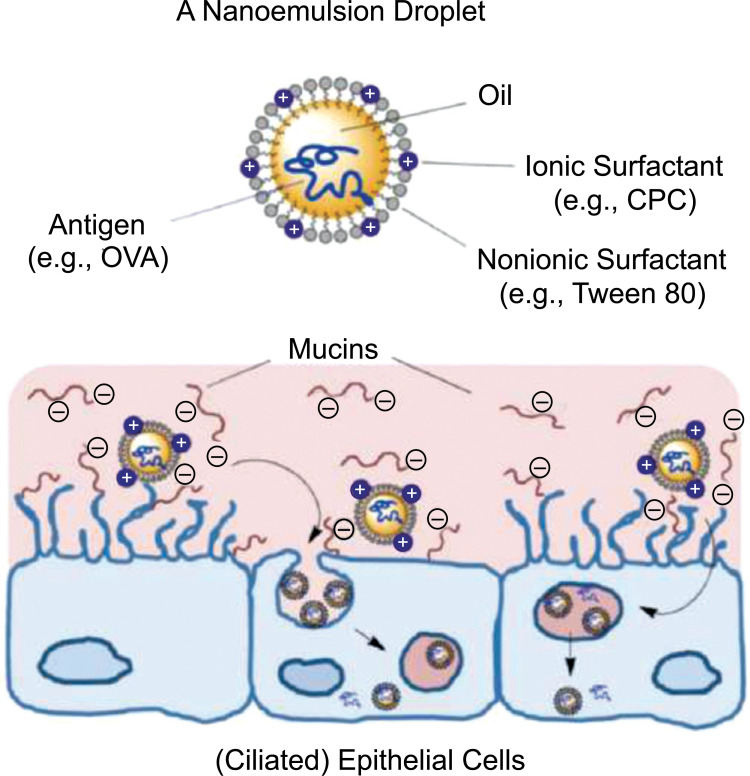
Structure of a nanoemulsion (NE) droplet and illustration of NE-mediated antigen delivery through the nasal epithelial layer as facilitated by NE−mucin interactions (cetylpyridinium chloride, CPC; ovalbumin, OVA). Reprinted from: Mol Pharm, 11(2), Wong PT, Wang SH, Ciotti S, *et al.* Formulation and characterization of nanoemulsion intranasal adjuvants: Effects of surfactant composition on mucoadhesion and immunogenicity. Page nos. 531-44, 2014, with permission from: ACS Publications [2].

Self-Nanoemulsification method allows avoiding the use of heat and organic solvent to generate nanoemulsions. The stepwise water addition into the solution of surfactant in oil under gentle stirring generates small droplet size (~50 nm) [66]. An antigen can be incorporated into the oil core of a nanoemulsion droplet. It is covered by surfactants and thanks to its characteristics and the emulsion surface, it can interact with the mucous layer and then the epithelial cells where it can be endocytosed (Fig. **2**).

The advantage of incorporating a vaccine into the nanoemulsion droplet is not only for physical reasons of compatibilities between the different charges since that oil-made adjuvant can be easily incorporated in the oil core of an oil-water nanoemulsion, but it is also considerable how the nanoemulsion helps with mucoadhesion and leads to longer retention in the nasal mucosa, antigen-adjuvant permeation through the mucous layer and cellular uptake [2].

#### 1.2.2 Polymer-based Nanocarriers

Other carriers that have been proposed for the delivery of antigens *via* mucosal routes are polymer-based and can be classified based on the polymer nature. Polyester nanoparticles: they are biodegradable polymeric particles made up of polyesters obtained, in most cases, from poly (lactic acid) and poly (lactic- co-glycolic acid) and their copolymers of variable molecular weight and composition. Polyester nanoparticles can transport bioactive macromolecules, including peptides, proteins, and nucleic acid-based vaccines [67, 68]. The presence of poly (ethylene glycol) (PEG) on the particle surface can lead to improved physical and biological stability through a steric stabilization mechanism given by the formation of a hydrophilic PEG shell around the particle core [69].

##### 1.2.2.1 Lectin-targeted Polymeric Nanocarriers

Lectins are structurally distinct proteins and glycoproteins with the property of reversibly binding to specific carbohydrates. Their bioadhesive properties enhance their affinity to mucosal surfaces. Targeting of antigens to antigen-presenting cells could be facilitated by lectin-mediated vaccine delivery [70]. Therefore, polymeric particles can be surface customised with lectins to enhance their immunogenicity.

##### 1.2.2.2 Polysaccharide-based Nanocarriers

They can be prepared from various polysaccharides such as cellulose derivatives, hyaluronate, pectin, alginate, and chitosan [71]. Among the polysaccharides, chitosan-decorated nanocarriers seem to be the most important for vaccine applications. Chitosan is a polysaccharide composed of N-acetylglucosamine and D-glucosamine units and can facilitate the mucosal transport of bioactive molecules (including antigens) thanks to its properties of bioadhesiveness and ability to induce the temporary opening of tight junctions. It is characterized by good biocompatibility and low toxicity, features that make chitosan one of the most investigated polysaccharides for vaccine mucosal delivery [72-74]. In Table **1** are summarized pros and cons of chitosan main chemical and physical characteristics.

Because chitosan is a bioadhesive carbohydrate, it may decrease the clearance of formulations from the nasal cavity in both animal models and humans [75, 76]. Besides simple solutions or powders, the chitosan has been formulated also in more sophisticated micro-and nanoparticulate formulations [77]. Nasal route administration is a route in which the vaccines administered remain in the nasal cavity mostly around 15 min and are not exposed to low pH values and degrading enzymes present after oral administration, furthermore, the vaccines administered have to be transported over a very small distance to get in contact with immunocompetent cells [78].

## 2 PATENTS

There are several different types of vaccines that can be administered through the nasal cavity. Those vaccines can induce humoral immune responses by using various types of antigens for each specific causative agent. Here below are reported examples of different vaccine patents using diverse adjuvants, formulations, and manufacturing techniques. Some aspects related to their nature, safety and advantages are summarized in Table **2**.

### 2.1 Liquid Compositions

**Fig. (3):**
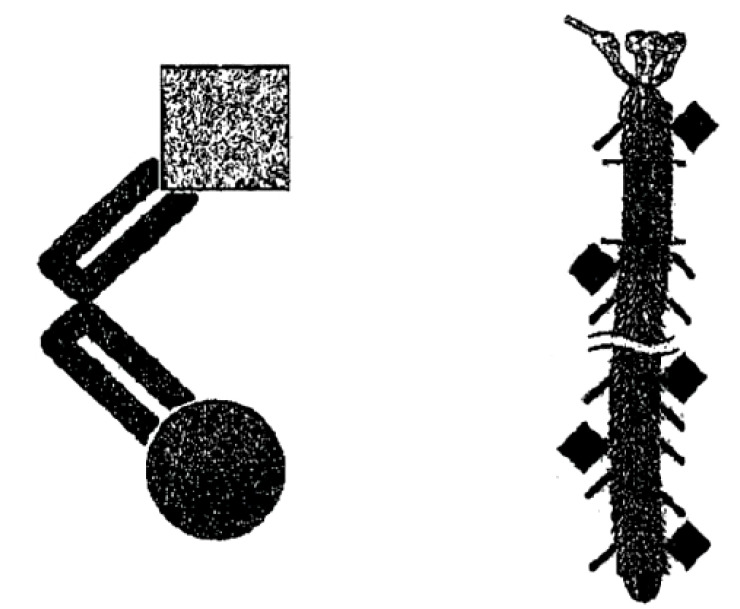
The image on the left depicts the molecular complex showing the “locomotive polypeptide” that can drive endocytic uptake and transport into CNS neurons, and even drug-binding polypeptide that can link and subsequently release a therapeutic drug. The image on the right depicts a phage particle that carries the “locomotive polypeptide” as well as additional adjuvants that can stimulate an immune response, for vaccines that can be administered *via* oral or nasal sprays [79].

Most patients are presenting nasal vaccines formulated in liquid form that is because these can be easily administered nasally by using metered spray devices. An example is the English Patent GB2460969 [79] that propose a molecular complex comprising a bacteriophage displaying a peptide that targets the nasal-associated lymphoid tissue (NALT) and an antigen for use as a vaccine for intranasal administration. This patent furthermore claims a method of selecting a phage comprising a peptide that targets NALT (Fig. **3** left panel).

The phage is a filamentous phage and the molecular complex contains an additional immunostimulatory adjuvant element, which is a DNA sequence that contains CpG motifs that can activate immune cell surface receptors (Fig. **3** right panel). The gene encoding the antigen is inserted into a coat protein of the phage.

Another example is Chinese patent CN102764430 [80] in which the inventors disclose a nasal spray mucosal vaccine composition and a method of preparation. The vaccine disclosed in this patent comprises the following components: an immunocytokine (0.01-20%) used as an adjuvant for the mucosal vaccine, and as an immunoregulator; a quaternary chitosan salt hydrogel (45-80%) used as a biological adhesive and as an antigen transfer system; the vaccine antigen (0.01-20%); thimerosal (0.01-15%); polyethylene glycol (0.5-2%), which serves as a stabilizer and to enhance the immune response of the antigen in the nasal cavity. Thanks to this formulation, the vaccine is effectively improved for the nasal mucosa and side effects such as irritation caused by the vaccine spray are reduced. It is also claimed that the ingredients of the vaccine work together synergistically.

The immunocytokine of choice is a natural or recombinant cytokine among the group of interleukins (IL-1α, IL-1β, IL-2, IL-3, IL-4, IL-11, IL-12, IL-13, IL-15, IL-17, IL-18, *etc*.). The immunostimulant antigen can be an inactivated or attenuated viral antigen, a bacterial antigen, a natural or recombinant protein antigen, a polysaccharide antigen, a nucleic acid antigen or one or more protein-binding polysaccharide antigens [80].

The Japanese patent JP2013253086 [81], is an example of an invention providing a method and composition for stimulating an immune response to an environmental pathogen by employing a nanoemulsion as a mucosal adjuvant that induces immunity. The present invention consists of a vaccine comprising an emulsion, and an immunogen, wherein the emulsion comprises an aqueous phase, an oil phase, and a solvent.

The immunogenic component used in the vaccine is a pathogen, a pathogen-derived component, or an inactivated pathogen. The immunogenic part is taken from a group of pathogens including viruses, bacteria, and fungi or products derived from viruses, bacteria, and fungi. This invention claims to use in the oil composition an oil selected from the group derived from soya, avocado, squalene, olive, oilseed, rape, corn, rapeseed, safflower, sunflower, fish, and includes water insoluble vitamins. The solvent is selected from the group consisting of alcohols, glycerol, polyethylene glycol, and organic phosphate-based solvents. The alcohol phase is composed of alcohol selected from the group consisting of methanol, ethanol, propanol, and octanol. In some embodiments, the therapeutic agents (*e.g.* vaccines) of the invention are administered in the form of a topical emulsion, an injectable composition, an ingestible solution, and the like. Where the pathway is local, the configuration may be, for example, a spray (*e.g.* a nasal spray) [81].

Patent US2010092526 [82] is another example of an invention related to therapeutic nanoemulsion composition and to methods of utilizing the same. The compositions described in this case are nanoemulsions that find use in the treatment and/or prevention of infections, such as respiratory infections (*e.g.*, related to cystic fibrosis), in the treatment of burn wounds, and in immunogenic compositions that elicit an effective immune response in a patient treated with the immunogenic composition. The invention described in the patent relates to methods and compositions useful for treating pulmonary infections. The nanoemulsion compositions described are claimed to treat bacteria associated with biofilms, in particular, those found in pulmonary infections, by one or more gram-positive or gram-negative bacterial species, such have those occurring in cystic fibrosis (CF) patients. The nanoemulsion composition comprises water, at least one organic solvent, at least one surfactant, and at least one oil. In addition, the droplet making up the nanoemulsion must have an average diameter of less than 1 µm. The bacterial antigens were selected from the group consisting of *Staphylococcus* spp., *Haemophilus* spp., *Pseudomonas* spp., *Burkholderia* spp., *Acinetobacter* spp, *Stenotrophomonas* spp., *Escherichia* spp., *Klebsiella* spp., and *Proteus* spp. The patent also describes specific compositions useful in the methods of the invention. Exemplary compositions include, but are not limited to, nanoemulsions comprising: (a) water; (b) ethanol or glycerol; (c) cetylpyridinium chloride (CPC), or benzalkonium chloride, or alkyl dimethyl benzyl ammonium chloride (BTC 824); (d) soybean oil; and (e) Poloxamer 407, Tween 80, or Tween 20. In another embodiment, the nanoemulsion may further comprise EDTA. The nanoemulsion described herein may be administered in any pharmaceutically acceptable manner, such as intranasally, buccally, sublingually, orally, rectally, ocularly, parenterally (intradermally, intramuscularly, intravenously, subcutaneously, intracisternally, intraperitoneally), pulmonary, intravaginally, locally, topically, *via* the mucous membranes, *via* an aerosol, or *via* a buccal or nasal spray formulation. In addition, the nanoemulsion vaccines above described can be formulated in some pharmaceutically acceptable dosage forms, such as gels, liquid dispersions, pulmonary or nasal aerosols, ointments, creams, semi-solid dosage forms, or as suspensions [82].

Another example of an invention regarding an oil-in-water nanoemulsion is the one presented in the patent CN105251002 [83]. This patent discloses an oil-in-water type nanometric emulsion adjuvants and methicillin-resistant *Staphylococcus aureus* (MRSA), nanometric emulsion adjuvant vaccine and the method for preparing it. Immunity can be achieved by various forms of administration such as nasal drops, but also by intramuscular injection. The adjuvant in the form of nanometric emulsion is prepared from 1-30% by mass of surfactant, 0.1-15% co-surfactant, 0.1-15% oil phase, and 40-98.8% water. The adjuvant prepared is a clear and transparent liquid emulsion with a droplet size of 1 nm to 100 nm and low viscosity. The adjuvant is a thermodynamically stable system so that high-speed centrifugations do not cause phase separation. The adjuvant is compatible with the vaccine or is directly mixed with the antigen. The adjuvant presents low cost, negligible toxicity, does not irritate the organism, is fluid, and convenient to administrate. To obtain a clear nanoemulsion an adjuvant vaccine is necessary to emulsify the oil phase by mixing at a temperature of 4-37°C, at the speed of 50 to 500 rpm; to add the protein antigen at the final concentration of 100 to 600 μg/ml in the mixed emulsion, and uniformly stirring at a temperature of 4 to 37°C and a rotation speed of 50 to 150 rpm; add distilled water to obtain a clear solution; add the remaining water to the clear solution obtained in the latest step and mix the nanoemulsion adjuvant vaccine obtained [83].

The patent JP2015091795 [84] presents how a nasal mucosal vaccine can safely prevent infectious diseases or even cancer, inducing both systemic immune response and mucosal immune response in human and animals. This nasal mucosal vaccine is composed of at least one kind of antigen (except for the influenza virus-derived antigen). As immunopotentiation agents are used, gram-negative bacteria such as *Serratia, Leclercia, Rahnella, Acidicaldus, Acidiphilium* to name a few, and lipopolysaccharide or a salt thereof derived from one of the gram-negative bacteria.

The specific lipopolysaccharide was selected as an adjuvant, by administering to the nasal mucosa with the antigen can induce the mucosal immune response. It is claimed that the nasal mucosal administration of the vaccine-induced the production of both IgG and IgA antibodies thanks to the induction of local immunity. The nasal mucosal vaccine composition object of this invention proposes a specific ratio between the total mass of the immunostimulatory agent and that of the antigen. This mass ratio of the immunostimulating agent and antigen may preferably be between 0.002 and 500. Below 0.002 immunity may not be induced, while for values above 500 safety concerns may occur. The antigen used in the composition is an infectious disease-derived antigen or cancer antigen, containing at least one kind of antigenic molecule and immunostimulatory agent. This invention cannot be used in the case of influenza-derived agents because the influenza virus mutates rapidly each year, so it would be necessary to produce a vaccine corresponding to each of the virus strains. The nasal mucosal vaccine composition mentioned in this invention is preferably a solution, spray, or a solid, or semisolid preparation. It can be administered to the nasal mucosa in both humans and animals. A spray device can be used to administer the vaccine composition to the nasal mucosa. In addition, other excipients, such as a binder, flavouring agent, sweetening agent, preservative, antioxidant, colouring agent, stabilizers, or surfactants can be used in the preparation [84].

The patent KR20050017770 [85] discloses an ionic nanoparticle carrier for the vaccine and a method for manufacturing the same. As carriers bind to the vaccine, the vaccine can be administered rapidly and with ease to the nasal mucosa. The preparation method of the ionic carrier is based on the following steps: dissolving biodegradable polymers in the organic solvent (1 to 4%w/v); preparing an anionic or cationic surfactant solution as an ionic emulsifier (1 to 8%w/v); sonicating the ionic aqueous solution and adding the biodegradable polymer solution; purifying the nanoparticle obtained by ultrafiltration. The ionic properties of the nanocarrier facilitate the delivery of the vaccine to the nasal mucosa and the preparation methods used for the ionic nanoparticles guarantee excellent stability for delivering a vaccine using inactivated viruses [85]. The patent US2015328296 [86] discloses a nasal vaccine against the development of atherosclerosis disease and fatty liver. This invention describes an innovative intranasal vaccine based on micellar nanoparticles for the treatment and/or prevention of atherosclerosis caused by the abnormal metabolism of circulating lipids. Since the inflammatory process is associated with atherosclerosis, innate and adaptive immunity are used in alternative treatments. Approaches have focused on modulating pre-existing immune responses to oxidised LDL (oxLDL) and heat shock protein 60 (HSP 60) or neutralizing several proinflammatory targets such as, cholesteryl-ester transfer protein (CETP) and TNF-a [87]. More particularly, the invention disclosed herein relates to a vaccine compound targeting cholesterol ester transfer protein (CETP) to prevent and halt the development of atherosclerotic coronary disease, which is reported by the World Health Organization to be the first cause of death worldwide. There are 476 amino acids that constitute the hydrophobic protein CEPT playing a key role in lipid metabolism [88].

As high-density lipoproteins (HDL-C) decrease and low-density lipoproteins (LDL-C) increase, this protein is overexpressed leading to atherosclerosis [89-91]. Therefore, a valid treatment option for the alleviation of atherosclerosis could be to inhibit the activity of CETP [91]. The fusion heat shock protein 65 kDa (Hsp65) of *Mycobacterium tuberculosis* var. bovis with the linear polypeptide epitope of the cholesteryl ester transfer protein C-terminal fragment (CETPC) and expressed as a soluble protein in Escherichia coli allowed to produce the Hsp65-CETPC vaccine [90]. CETP contains neutral lipids and since the basic principles that allow neutral lipids to reach the core tunnel have not been understood yet, García-González *et al*. have proposed a lipid ordering mechanism based on the formation of micelle-like structures supported by secondary structural transitions at the amphipathic carboxy-terminal CETP region. In accordance with thermodynamic parameters, the newly developed mechanism would allow lipids to move through an aqueous interface [92]. The α β-sheet (AA 453–462) and the native amphipathic α-helix (AA 465–476) named helix-X^12^ compos C-terminal region of CETP (aa 453–476); when glutamic acid 465 is replaced by a cysteine residue, special secondary structure properties develop with this new model peptide known as helix-Y^12^. When the peptide helix-Y^12^peptide is placed in solution and incubated at various pH values, it retains most of its -helical structure within a wide range of pH values (6.3-9.5) when examined by circular dichroism (CD) [86, 90, 92-94]. The aggregates of 30 nm formation have been demonstrated by dynamic light scattering (DLS) when phosphatidylcholine and cholesteryl esters were mixed under the same pH range. The large aggregate formation has been confirmed by negative staining transmission electron microscopy (NS-TEM). The formation of small homogeneous micelles with an average size of 6 nm is induced when the same lipid mixture is incubated together with peptide helix-Y^12^at a pH of 7.0. [93, 94]. While, at pH 6.3, part of the sample remains as large aggregates, and part as small micelles. This occurs because the peptide helix-Y^12^ has a net charge of -0.6 at pH 6.3, which is not consistent with optimal conditions for peptide-lipid interaction. Aggregates are formed at pH values about the isoelectric point for both helix-Y^12^ (5.09) and helix-X^12^ (4.17). Thus, the ability of the peptide to promote the organization of micellar lipid structures is only evident when the helix-Y^12^ peptide is tested at physiological pH values. Indeed, this pH allows the stabilization of the carboxy-terminal amphipathic α-helix. The helix-X^12^ peptide also performs the lipid order phenomenon, although to a lesser extent [92]. The use of lipid Archaebacteria, lysophosphatidylcholine, and phosphatidylcholine are the novelty of the vaccine of the present invention. These properties confer stability to the nanoparticles and facilitate the presentation of the antigen in its appropriate secondary peptide conformation. The characteristics of the archaebacterian lipids permit a system which unlike other adjuvants, provokes minor side toxic effects, while maintaining the stability of the formulation and improving the absorption in the mucosa. The lipid structure of archaebacterian lipids composed of long-chain alkyl groups bound by ether bonds with glycerol is a characteristic that allows these molecules to have scaffolding functions in micellar nanoparticles, which, at the same time determines the size and increases in an important manner the stability of such micelles. The process for the preparation of vaccine compounds allows for obtaining highly stable and homogeneous nanoparticles [93].

**Fig. (4):**
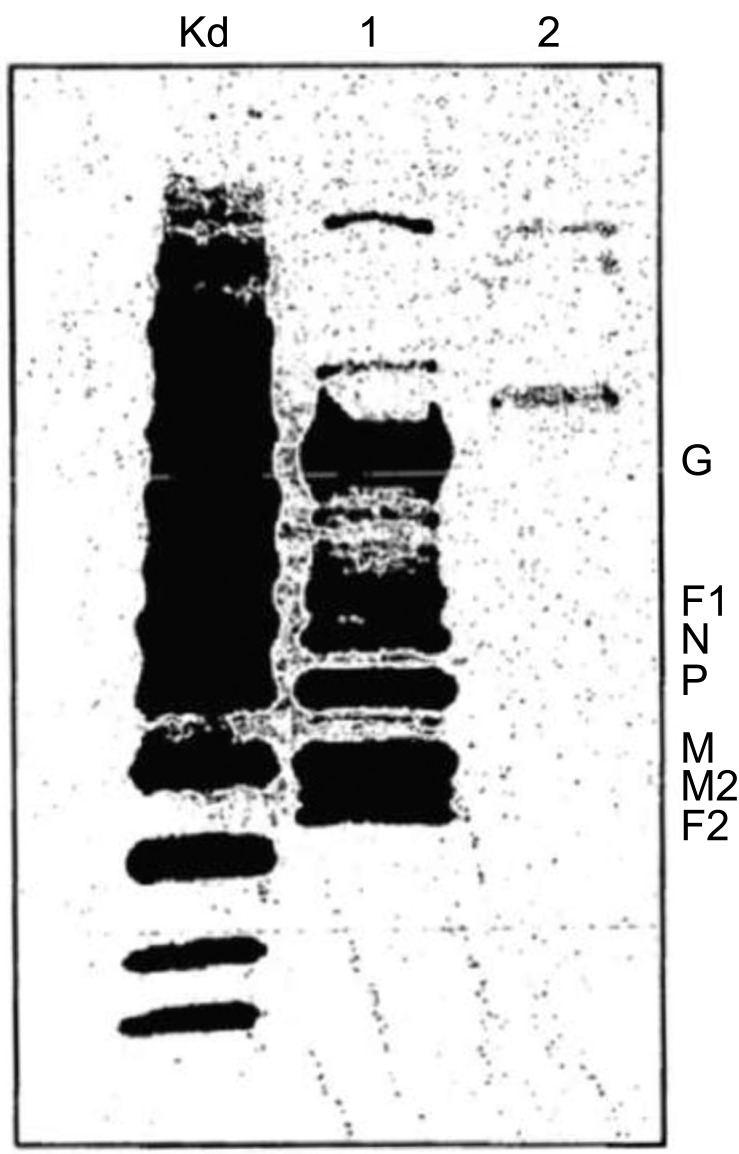
The expression of RSV cDNAs following intranasal GXV vaccination; immunoblot analysis. Lane Kd is a molecular weight marker; Lanes 1 and 2 are RSV infected and uninfected HEp-2 cell extracts [95].

The patent US2007009951 [95] is an effective prophylactic mucosal gene expression vaccine (GXV) consisting of a cocktail of at least 4 different plasmid DNAs encoding the corresponding respiratory syncytial virus (RSV) antigens. It is coacervated with chitosan to form nanospheres. The vaccine was developed for intranasal administration and tested in a mouse model of RSV infection. Intranasal administration of GXV results in significant induction of RSV-specific antibodies, cytotoxic T lymphocytes, nasal IgA antibodies, and IFN-gamma production in the lung and spleen cells (Fig. **4**). To obtain a dramatic reduction in viral titer a single dose of GXV is enough. The claim is to provide an effective vaccine that confers protection to a host against the respiratory syncytial virus (RSV)-induced disease. More specifically, the invention relates to gene expression vaccines that can be administered intra-nasally or orally. It has been demonstrated that the proteins N, SH, F, M, M2 and NS2 are potent target antigens of CTL repertoire in humans [95].

The invention in the patent CN105342982 [96], discloses an influenza vaccine immune preparation for nasal administration and a preparation method thereof. The influenza vaccine immune preparation for nasal administration contains the following ingredients: valent inactivated influenza vaccine, an immunopotentiator, osmotic pressure regulator, pH regulator, and water. The immunopotentiator is a liposome prepared from soy lecithin, and cholesterol in absolute ethanol heated in a 50°C water bath to allow dissolution. Finally, the inactivated influenza vaccine with HA content was dissolved in phosphate buffer (pH=7.0) and mixed with the lipid membrane to obtain a liposome suspension containing the influenza vaccine.

The invention has the following advantages: the developed influenza vaccine immune composition for nasal administration does not require injection administration, and the immune effect of the influenza vaccine immune composition is similar to the intramuscular injection effect [96].

The invention in patent CN102580083 [97] relates to a method for preparing a nanoparticle oil adjuvant vaccine. The formulation was prepared as follows: to obtain a solution A, a chitosan solution was added to the liquid solution of the Newcastle disease virus; adding sodium tripolyphosphate and phosphate buffer solution (PBS) into the solution A was obtained solution B. The obtained final solution was centrifuged and sterilized to carry out a white oil for injection. The nanoparticle oil adjuvant vaccine is small in particle size of virus-loaded nanoparticles, high in envelop rate, and large in medicine-carrying quantity, and the method has a mild preparation condition and a simple preparation process and is low in production cost [97].

The US2017007689 [98] claims a novel nanoemulsion vaccine composition and methods of making treating, preventing, or protecting an individual from anthrax exposure or poisoning. The present disclosure relates primarily to the methods and composition of vaccine formulations comprising stabilized recombinant protective antigen (rPA) of anthrax, a nanoemulsion, and a stabilizing system, where the stabilizing the system comprises a TRIS buffer, salt, sugar, and amino acid. The nanoemulsion (mean droplet size 400 nm) is W805EC adjuvant at a concentration of 20%. All components of the nanoemulsion are on the FDA list of inactive ingredients for approved drugs. The nanoemulsion vaccine adjuvants are formed by emulsifying oil, purified water, non-ionic detergent, organic solvent, and surfactant, such as a cationic surfactant. The “60% W805EC” is a specific vaccine adjuvant composed of: purified water, soybean oil, dehydrated alcohol, Polysorbate 80 NF, and cetylpyridinium chloride. The simple classical techniques used to prepare emulsions are also suitable for the excipients of nanoemulsions. To obtain a nanoemulsion with oil droplets of a mean diameter of less than 1 µm, the aqueous phase is mixed with the oil under high shear forces (hydraulic and mechanical forces) using high shear mixers or the French press. Sometimes some nanoemulsions contain ethanol in the oil phase. Methods of producing such emulsions are described in U.S. Pat. Nos. 5,103,497, and 4,895,452 [99].

The composition for mucosa and the method for producing the same is explained in patent JP2014129330 [100] which is related to a composition of mucous membranes in which the nanoemulsion containing a fat-soluble active ingredient is blended. Active ingredients such as fat-soluble vitamin A are attracting attention as effective components for the treatment of cornea, conjunctiva, nasal mucosa, and pharynx. However, these active ingredients are extremely sensitive to air, light, heat, acids, metal ions, and the like, and are extremely unstable, particularly in aqueous solutions. Parameters such as the setting of optimum pH, nitrogen filling in the pillow, combination with antioxidants, container colour, *etc*. have been considered for stabilization of these active ingredients, for example, vitamin A, in solution. Adding surfactant (necessary for emulsification of fat-soluble ingredients such as vitamin A) and, in some cases, an absorption promoter. The mucosal composition contains (A) a fat-soluble active ingredient in an amount from 0.001 to 1.0 w/v% and (B) a nonionic surfactant in a mass ratio represented by (A):(B) in an amount of 1:0.4 to 24.0. The oil phase containing the above components (A) and (B) are added to an aqueous phase at high temperatures. The particle size of these nanoemulsion particles is 1 nm or more and less than 500 nm. As the fat-soluble active ingredient, it included vitamin A-containing mixtures such as vitamin A oil, and vitamin A derivatives such as vitamin A fatty acid esters, in addition to vitamin A per se. Some preferable examples are retinol palmitate, retinol acetate, and retinoic acid. The fat-soluble active ingredient other than the fat-soluble active ingredient (A) is not particularly limited but includes antioxidants such as tocopherol acetate, which may be used singly or in a combination of two or more. The non-ionic surfactants used include a non-ionic surfactant selected from the following polyoxyethylene hydrogenated castor oil and polyoxyethylene sorbitan fatty acid ester, polyoxypropylene copolymer. As is shown in the described examples of this patent, it is possible to maintain the stability of the vitamin A, make the appearance of the mucosal composition homogeneous, and increase the adsorption of vitamin A (*i.e.,* vitamin A adsorption rate of 40% or more to the cornea) [100].

The invention in patent US2017360919 [101] provides for a novel formulation of respiratory syncytial virus (RSV) surface antigens. This novel vaccine claims that the mixture of F and G proteins in the nanoemulsions overcomes the inadequate immune response observed in previous RSV vaccine data [102]. The average droplet diameter of RSV subunit vaccine in nanoemulsion is less than 1 µm. The presence and presentation of an optimal level of antigens induce enhanced immunity to RSV. Namely, the novel approach of combining isolated RSV surface antigens with nanoemulsion provides appropriate antigen-presenting cells for the immune response. In this RSV formulation, the nanoemulsion is the vaccine adjuvant. Adjuvants can play several roles. First and foremost, they play a major role as carriers of the antigen that stimulates the protective immune response also in terms of quality (duration or magnitude). Adjuvants potentially reduce the toxicity or required amount of some antigens, thereby reducing the number of doses required. Some adjuvants can increase the solubility of vaccines. To evaluate the immune response of the subject after administration of the RSV nanoemulsion vaccine, the titer and/or presence of antibodies to the RSV immunogen should be determined. To evaluate the protective immune response, seroconversion, for example, allows the quantification of specific antibodies to an immunogenic measured by Western blotting or enzyme-linked immunosorbent assays (ELISA) or haemagglutination inhibition (HAI) tests. A safe and effective nasally administered vaccine against hepatitis B is that described in Patent US2017360919 (A1), in which a nanoemulsion has been added to the hepatitis B surface antigen (HBsAg) [101].

The patent CN102688488 [103] provides a mucosal adjuvant prepared by copolymerizing cross-linking oligo-chitosan and lymphotactin encoding plasmids. A method of preparation and the use of the mucosal adjuvant are also disclosed. The invention also discloses a preparation method and the use of the mucosal adjuvant. When the mucosal adjuvant and gene vaccine composed of chitosan or oligo-chitosan are synchronously dropped into the nasal cavity for immunization, coxsackievirus specific serum antibodies and systemic (spleen and lymph glands) Th1-type immune responses especially, intestinal mucosa partially-reinforced specific-secretion-type sIgA and IFNγ+Th1 responses are induced, and thus a gene vaccine used with the mucosal adjuvant is clearly superior to an exposed gene vaccine and effectively prevents coxsackievirus-caused myocarditis. The mucosal adjuvant can be used as a novel adjuvant for mucosal vaccination. The method for preparing the above mucosal adjuvant comprises the steps of: constructing a mouse-derived plasmid encoding lymphocyte chemotactic factor encoding plasmid; a step of preparing a chitosan oligosaccharide solution having a concentration of 0.1 to 0.4% and a pH of 5.2 to 5.5. The above chitosan oligosaccharide solution and a plasmid DNA solution encoding lymphocyte chemotactic factor dissolved in a buffer at a concentration of 18-25 μg/ml are mixed at a high speed at 60°C and passed through copolymerization cross-linking results in a mucosal adjuvant having a spherical shape and a diameter of between 250 and 350 nm [103].

The invention in Patent CN102370977 [104] discloses a novel vaccine adjuvant (also called an SPO1 vaccine adjuvant) and a preparation method thereof. The vaccine adjuvant disclosed by the invention mainly comprises squalene, polyoxyethylene, castor oil, and polyether, and can realize immunization through injection or nasal spray, transdermal non-injection, and other approaches; and the vaccination through non-injection has the characteristics of low cost, convenience for administration, avoidance of cross infection, and high safety. The SPO1 vaccine adjuvant disclosed by the invention can be used for preparing vaccines along with inactivated whole pathogens, extracted components of cracked pathogen, bacterial vesicles, and capsular polysaccharides or polysaccharide-binding proteins, recombinant proteins, recombinant VLP (Virus-like Particle), DNA (Deoxyribonucleic Acid) or RNA (Ribonucleic Acid) and other different types of antigens, can be suitable for people in different age groups and preparation of different animal vaccines and immunization, has the advantages of no complex structure, simple process, convenience for preparation and sterilization, low cost and suitability for mass production, and industrialization. The preparation method of the vaccine adjuvant of the invention is: the raw material is prepared by using a homogenizer to prepare colostrum. After preparing a milky white vaccine adjuvant preparation, the buffer was selected from one of deionized water, physiological saline, phosphate buffer, sodium citrate buffer, and sterilized by filtration. The method for measuring the particle size of the colostrum, the homogenized colostrum, and re-homogenization is a conventional measurement method. The vaccine adjuvant of the invention may be a colloid or microsphere formed by a solution, the particle size is uniform, the particle size is between 90 nm and 200 nm, and the average particle diameter is about 150 nm; the oil-in-water vaccine adjuvant of the invention can be used with various antigens. Different types of vaccines are prepared by mixing whole pathogens, lytic pathogen extracting components, bacterial vesicles, capsular polysaccharide or polysaccharide binding proteins, recombinant proteins, recombinant virus-like particles (VLPs), DNA or RNA nucleic acids, *etc*., wherein the vaccines are according to conventional techniques. A conventional excipient is added to prepare various dosage forms such as pharmaceutically acceptable injection, microneedle matrix agent, nasal spray, transdermal patch, and the like, wherein the vaccine is suitable for immunization of different age groups and different animals. The invention is a safe, effective, and stable SPO1 vaccine adjuvant, which can recruit immune cells and immune molecules to aggregate at the antigen injection site or mucosa site, enhances the effect of specific cellular immunity and humoral immune response and can adjust the affinity of the antibody to the antigen. At the same time, it can be immunized by injection or mucosal, and mucosal vaccination has the characteristics of low cost, convenient administration, avoiding cross infection, and high safety compared with vaccination immunization. SPO1 vaccine adjuvant can be applied to special groups such as children, the elderly, pregnant women, and various animals, and has no complicated structure, or simple process, and is suitable for mass production industrialization [104].

The invention in patent CN101695567 [105] discloses a water-in-oil type nanoemulsion vaccine preparation, which is prepared from the following raw materials in percentage by weight: 10 to 70 percent of oil, 20 to 60 percent of a surfactant, 5 to 30 percent of cosurfactant, 1 to 40 percent of antigen, 0.01 to 40 percent of immunopotentiator, wherein the total weight percentage of the raw materials is 100 percent. The nanoemulsion formulation cannot not only improve the stability and safety of the vaccine and reduce the side reactions of the vaccine but also can remarkably improve the humoral response and cell response to the antigen and reinforce the immunization of mucous membranes of organisms. The nanoemulsion vaccine prepared by the invention is an oil-in-water type nanoemulsion, and the appearance is clear and transparent, and observed under a transmission the electron microscope, the nanoemulsion particles are spherical, evenly distributed, and have no adhesion. The oils in the nanoemulsion of the invention include mineral oils such as soybean oil, malt oil, squalene, and light liquid paraffin, ethyl acetate, IPM (isopropyl myristate), and the like, which can reduce the body of the animal. In a further aspect, the invention relates to the use of the vaccine nanoemulsion in preparing an immune vaccine, wherein the antigen involved comprises an inactivated pathogen such as Newcastle disease inactivated antigen, avian influenza inactivated antigen, and blue ear disease inactivated antigen. The vaccine preparation of the present invention has the following advantages and effects as the existing commercial vaccine: It has unique stability and can be placed at 40°C for 40 days without delaminating or demulsification; low viscosity for easy injection; mucosal inoculation, no vaccine side reactions; the immune effect is ideal, and humoral immunity and cellular immunity can be induced simultaneously. Moreover, the vaccine has a long period of immune protection and the preparation cost is low and has broad market prospects [105].

### 2.2 Gel, Semi-solid Composition

The following examples of patents report formulations in the form of gel or semisolid preparations. Thus, a patent disclosing a cationic nanogel for mucosal vaccination is the Japanese patent JP2010105968 [106]. The invention aims to provide a mucosal vaccine for oral or nasal administration able to induce an immune response specific to a vaccine antigen in a living body without adding a mucous membrane adjuvant. The mucosal vaccine preparation can be administered through a mucous membrane and it is for preventing or curing an infectious disease caused by microbes. It contains a complex between the vaccine antigen and a nanogel formed by adding hydrophobic cholesterol as a side chain to a hydrophilic polysaccharide having a cationic amino functional group. The antigen is a microorganism-derived antigen and the microorganisms are selected from groups of viruses, bacteria, protozoa, and fungi. The molar ratio individuated to be optimal between the vaccine antigen and nanogel is from 1:1 to 1:10 [106].

Another representative example of this type of preparation is described in the US patent US2017014338 (A1) - 2017-01-19. This is an invention which is described as a nasal vaccine for *Streptococcus pneumoniae* formulated as a nanogel. The antigen of this nasal vaccine is a pneumococcal surface protein complex (PspA) and, together with the nanogel in which hydrophobic cholesterol is added as side chains to the pullulan amino groups, forms the formulation. *Streptococcus pneumoniae* is a pathogen similar to the influenza virus and clinically relevant, sometimes starting as an upper respiratory tract infection. *Streptococcus pneumoniae* of any type colonizes the nasal cavity and causes an initial infection of the respiratory tract mucosae, thus a nasal vaccine is expected to be the most effective method for preventing *Streptococcus pneumoniae* infection. However, there is currently no safe adjuvant for nasal immunization or delivery system of the nasal vaccine, as evaluated by the so-called safety pharmacology studies. Furthermore, when biologically active mucosal adjuvants such as cholera toxin (CT) and heat-labile enterotoxin (LT) are administered simultaneously, there is concern that the toxin may enter the central nervous system or accumulate in the olfactory bulbs and the like. To address these concerns, Yuki *et al*. recently developed a vaccine composed of a self-assembled nanometer hydrogel (nanogel) composed of a cationic type of pullulan with cholesterol groups (cCHP). The cCHP nanogel retains antigen proteins in its nanomatrix and acts as an artificial chaperone that prevents aggregation and denaturation of antigens and supports refolding after release. As an adjuvant-free vaccine, cCHP nanogel is efficiently transported to cells and induces immune responses [106-111].

### 2.3 Solid Compositions

In this section, some examples of nasal vaccines made in solid form as dry powders or nanomicro spheres are presented.

The patent US5942242 [112] describes the invention of a nasal formulation in the form of a suspension or powder that releases a vaccine or pharmacologically active peptide through the mucosa and includes a powder of one or more exchange resins or multiple adsorbent resins. The carriers of the vaccine or peptide are powder particles (about 200 nm) in which the cation exchange resins are selected from the group consisting of sulfonated polystyrene, acidic polystyrene copolymers, and salts thereof. The vaccine used in this study is a pathogen selected from influenza, pertussis, diphtheria, measles, rubella, Japanese encephalitis, Weil's disease, cholera, mumps, chickenpox, viral hepatitis, tetanus, and Bacille Calmette-Guerin (BCG). The active peptide is selected from the group consisting of peptide hormones, physiologically active proteins and enzymatic proteins (such as insulin, calcitonin, elcatonin, somatropin, and glucagon). In addition, from the group consisting of buffers, smoothing agents, water-soluble polymers in powder form, fats, polyhydric alcohols, powdered sugars, powdered amino acids, water-soluble acids, bases and their salts, fragrances and chelating agents, the excipients contained in the above-mentioned medicament are selected [112]. Fig. (**5**) presents the serum insulin concentration over time produced by the nasal administration of medicament compositions described by Yutaka *et al.* as compared with that produced by the traditional injection method.

Another example of the mucosal vaccine in solid form is provided by patent CN104208029 [113] that discloses a vaccine in the form of a powder preparation used for nasal administration and a preparation method thereof. The vaccine is a powder composed of an antigen, an oil phase, a surfactant, a mucous membrane adhesion agent, and a freeze-drying excipient. The antigen (0.05-5%) was selected from an influenza virus antigen, hepatitis B virus antigen, or tetanus virus antigen. The oil phase is in the percentage from 0.3 to 45% and it is selected from the group consisting of squalene, squalene, medium chain triglyceride, fish oil, olive oil, and soybean oil. 0.3 to 45% of the preparation is provided by surfactant. The mucoadhesive agent is 2 to 20% and it is selected from the group consisting of chitosan, sodium carboxymethylcellulose, sodium alginate, carbomer and hydroxypropyl methylcellulose. A percentage between 4 and 40% is due to the lyophilized excipient and it is selected from lactose, sucrose, trehalose, insulin, amino acids, and mannitol. The powder is prepared by the following methods: the first step is mixing the oil phase and the surfactant together, after that, it is necessary to stir and add water to obtain the adjuvant. The following step is mixing the prepared adjuvant with the antigen and submitting the preparation to freeze drying to obtain the powder. This vaccine composition powder preparation is supposed to be administered by the nose for mucosal immunization. The vaccine composition powder preparation for nasal mucosa immunization shows good application prospects as the preparation method is simple, the vaccine stability is high, the administration is convenient, and the inoculation rate can be greatly increased [113].

The authors in patent JP2009209086 [114] disclose a mucous membrane administration-type vaccine. The invention relates to a mucosal administrable vaccine, which effectively induces the production of secretory IgA and/or IgG antibodies specific to viruses by administering the vaccine to the mucous membrane, particularly a respiratory mucosa such as a nasal mucosa in a resident manner. The presented mucosal administered vaccine is a powder vaccine containing a virus-derived inactivated antigen, an adjuvant, and a thickening agent, and can be used as a vaccine for mucous membranes, particularly respiratory mucosa. This vaccine has been prepared to be administered to the mucous membrane and contains an inactivated antigen originating from a pathogen, an adjuvant of a double-stranded or single-stranded RNA of a ligand of TLR (Toll-Like Receptor), chitin, chitosan or fine powder of *Spisula sachalinensis*, and a thickener. The thickening agent is selected from the group consisting of sodium alginate, propylene glycol alginate ester, carboxymethyl cellulose, calcium, sodium carboxymethyl cellulose, sodium starch glycolate, sodium starch phosphate ester, methylcellulose and sodium polyacrylate. The pathogen is selected from the group consisting of varicella virus, measles virus, mump virus, poliovirus, rotavirus, influenza virus, adenovirus, herpes virus, and severe acute respiratory syndrome (SARS) virus to name a few. The mucous membrane administration-type vaccine is claimed to have a prolonged residence time on the mucous membrane. It induces the secretion of the IgA antibody at the site and the IgG antibody response in the serum [114].

**Fig. (6):**
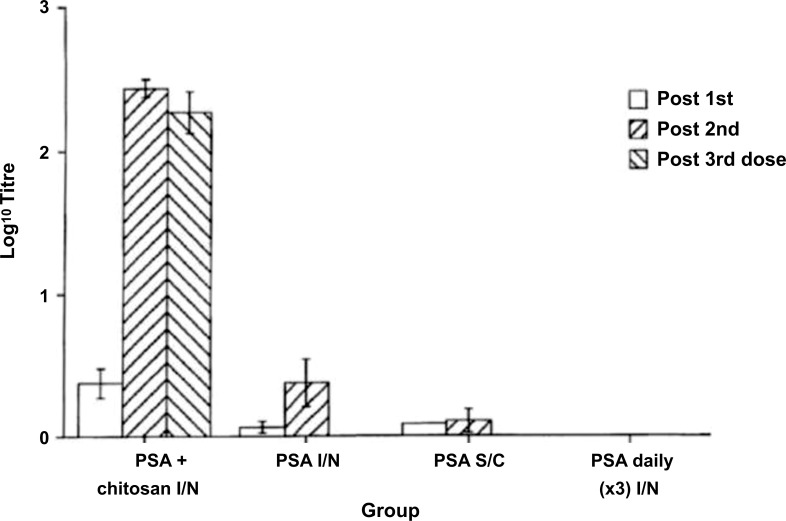
Illustrates the serum IgG anti-haemagglutinin response in mice immunized with the purified surface antigen of influenza (PSA). The cut-off value is 50, which is the lower limit of detection [115].

**Fig. (7):**
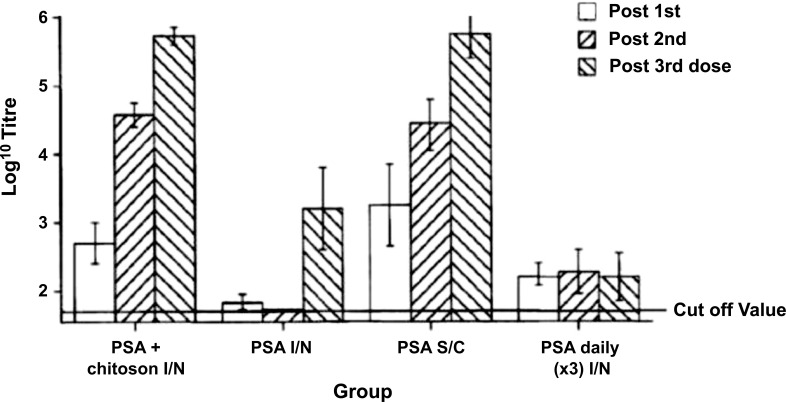
Illustrates the nasal IgA anti-haemagglutinin in response in mice immunized with the purified surface antigen [115].

Another example is Patent US6391318 [115] that disclose a vaccine composition including chitosan for intranasal administration and the use thereof. This invention is directed toward a novel nasal vaccine composition that utilizes the cationic polysaccharide, chitosan, as a delivery system (a polysaccharide comprising copolymers of glucosamine and N- acetylglucosamine). There are various chitosan polymers with varying molecular weights from 50 kDa to 2.000 kDa and degrees of acetylation (40%-98%). Several animal vaccine studies have been conducted using influenza or pertussis antigens in combination with chitosan. Nasal administration of chitosan antigen nasal vaccines induced significant serum IgG responses (Fig. **6**) and secretory IgA levels (Fig. **7**). Nasal vaccines are preparations of antigenic material administered to recipients to increase resistance to infection by inducing active immunity to the specific microorganisms, such as bacteria or viruses [115].

Vaccines can be presented as consisting of either inactivated whole virus, disrupted virus (split vaccines), or purified preparations of antigenic proteins. It has now been found that chitosan, when administered intranasally, enhances the immune response to antigens and thus has an adjuvant effect and elicits good systemic and local immune responses. In particular, Illum and collaborators claimed an intranasal vaccine in liquid or dry form (microspheres) that can be administered by aerosols, drops, or insufflations and that enhances both a protective IgA mucosal immune response and an IgG systemic immune response [115].

The patent “Composition and manufacturing of powders containing nanoadjuvants for mucosal vaccination” [116] claims the invention of a dry powder vaccine to be administered nasally or to any mucosal tissue for the prevention of infectious diseases. The proposed formulation is characterized by a liquid vaccine dispersion mixed with a submicronic adjuvant, which is a polysaccharide stabilized O/W nanoemulsion. The solid component of this vaccine can be varied with soluble or insoluble adjuvants depending on the purpose to be achieved. The α-tocopherol and/or sunflower oil as the oily phase, in combination with the surfactant PEG660 12-hydroxystearate and with low molecular weight chitosan as the stabilizing polysaccharide form the adjuvant. The solid carrier is calcium carbonate and the antigen is an inactivated whole cell concentrate of *Mycoplasma hyopneumoniae*. During the vaccine manufacturing, the antigen is finely dispersed on and firmly bound to the carrier without the need to resort a high potency compounding methods (*e.g*., the use of high temperatures or high energy mixing) that could reduce antigen activity. Despite the solid antigen-carrier aggregation, the product rapidly dissociates and releases the antigenic component when reconstituted in an aqueous environment, *e.g*., on a mucosal surface, allowing the rapid release of the particulate vaccine components. The efficacy of the vaccine was *ex vivo* and *in vitro* evaluated by the frequency of *Mycoplasma hyopneumoniae*-specific INF-γ secreting cells (SC) and memory B-cell responses in peripheral blood mononuclear cells. The efficacy of the dry powder vaccine of the invention was confirmed by *in vivo* study in terms of induction of systemic immunity to an extent equivalent to conventional intramuscular immunization with the same antigen. As shown in Fig. (**8**), the proportion of *Mycoplasma hyopneumoniae* myo-specific IFN-γ secreting cells SC was not significantly different in pigs when comparing intranasal vaccination with the dry powder vaccine of this patent and a conventional immunization with the same antigen [116].

The invention explained in patent AU2012244077 [117] claims methods for generating dry vaccine powder formulations that can be used for intranasal delivery and represent a method for stimulating local mucosal and systemic immunity. The present disclosure provides methods for preparing a vaccine, which can preserve a part or whole three-dimensional configuration of the antigenic component (*e.g.,* virus, protein). One of this methodologies can see the antigen (shown as an open circle) combined with a stabilizer (*i.e.,* trehalose) and a buffer (preferable phosphate buffer). The components are mixed and freeze-dried. The dried vaccine component produced comprises fine particles in which the antigen or antigens are still capable of eliciting an immune response and is stable at room temperature. The vaccine component can then be combined with a carrier suitable for nasal administration (*e.g.,* microcrystalline cellulose). The antigenic stabilizing agent will be wholly or partially water-soluble and is the preferred one that will not produce hard cakes in the processes of freeze-drying such as sugars, amino acids, polymers, and surfactants. The ratio of antigen to stabilizer has from 1:1 to 1:1000 while the pH of the vaccine liquid formulation used in the freeze-drying step is maintained between pH 3 and pH 8 with the addition of buffer phosphate. The liquid formulation is used to generate a powder formulation by the methods described in the patent can contain one or more other drugs, bulking agents, and/or sustained release polymers and it could be made without including an adjuvant. Thus, the final vaccine can be produced using only the pathogen/antigen, a stabilizer, and a buffer, which is then freeze-dried. The liquid formulation can be converted to a powder by freeze-drying. The method of freezing can prevent the loss of the three-dimensional shape of an antigen in the liquid vaccine formulation. The liquid vaccine formulation can be exposed to a cold liquid, *e.g*., liquid nitrogen, for about 30 seconds to 60 minutes. After quick freezing, the frozen formulation is freeze-dried in a freeze-dryer. This process can occur in one or more steps (*e.g*., different temperatures at the same pressure). The powder obtained can be stored (preserved) at a temperature of about 4 to 25°C. At last, the powder has a particle diameter size of about 5 to 100 µm and a low hygroscopicity. The formulation can be administered intranasally to stimulate the mucosa-associated lymphoid tissue (MALT), which can play a role in mucosal immunity, to stimulate the production of the principal antibody of the mucosal immune system, secretory IgA (sIgA) In addition; dry powder formulations have shown the potentiality to stimulate an IgG response, leading to an additional layer of protection. Thus, in one embodiment, the vaccines disclosed in this patient can induce both mucosal and humoral antibody responses [117].

## CONCLUSION

Nasal administration of vaccines is an attractive alternative because of the large number of immunocompetent cells present in the nasal mucosa. Mucosal vaccines represent an interesting alternative for targeted immunization against diseases in which pathogens enter the body through these epithelia. The ease of self-administration and the induction of mucosal as well as systemic immunity are the main advantages of nasal administration of vaccines. In addition, both liquid and powder formulations can be administered intranasally. In most cases, Nanometric adjuvants are not only a viable approach to improve the efficacy of nasal vaccines but represent the core intellectual property of the innovative vaccine.

## CURRENT & FUTURE DEVELOPMENTS

The nanoadjuvants exhibit great potential for nasal application of vaccines. It is expected that the interest in these nanoadjuvants will grow with the introduction to the market of more products adopting nasal administration as the election route for vaccination. Although various nanovaccine vectors have been introduced as potential immune factor adjuvants, further improvements are still needed before they can be brought to clinical use.

## Figures and Tables

**Fig. (5):**
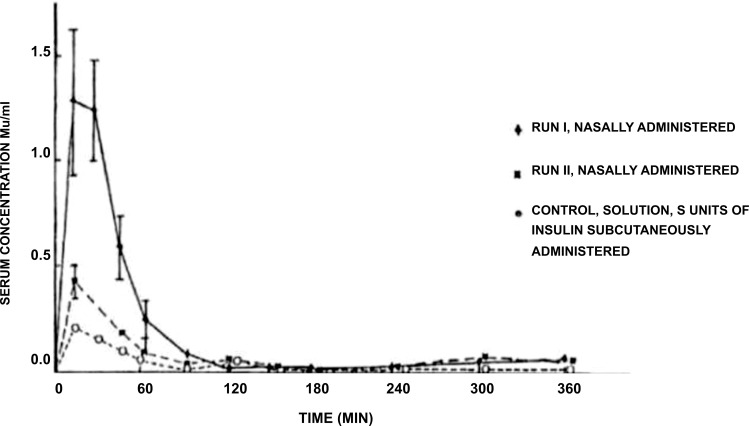
Insulin serum concentrations obtained by the nasal administration in rabbits of the compositions disclosed in reference [112].

**Fig. (8):**
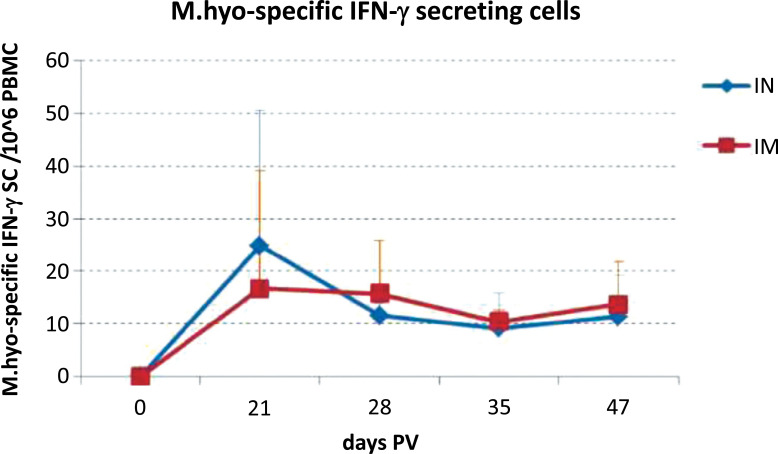
The fraction of *Mycoplasma hyopneumoniae* Myo-specific IFN-g secreting cells SC in pig comparing the patented powder intranasal (IN) vaccination with a traditional intramuscular (IM) immunization with the same antigen [116].

**Table 1:** Analysis of physicochemical and biological properties of chitosan used for delivery of biotechnology drugs [73].

**PROS**	**CONS**
Natural polymer	Low solubility at physiological pH (7.4) or higher pH values because it is a weak base (pK_a_ 6.2–7)
Readily available	High viscosity
Biodegradable and biocompatible	Contradicting data about its immuno-stimulating properties
Safe (low toxicity profile)	-
Forms salts with organic and inorganic acids	-
When protonated, adheres to negatively charged surfaces (bio/mucoadhesive), and forms gels with polyanions	-
Transmucosal absorption enhancer with minimal and reversible membrane damage	-
Amenable to chemical modifications	-

**Table 2:** Type, size, safety, and advantages of the different types of adjuvants presented.

**Type of Adjuvant**	**Size/weight**	**Safety**	**Advantages**	**Refs.**
**NALT targeting Bacteriophage**	24-200 nm	Low risk of systemic toxicity	Low-cost mass immunization	**[** **80** **]**
**Immunocytokines (IL)**	12-15 KDa	No toxicity reported	Improved retention time	**[** **81** **]**
**O/W Nanoemulsions**	< 1000 nm	Minimal or no toxicity	Synergistic effect with antibiotics for bacterial infection treatment. No resistance. Improved humoral and cell response. Reinforced immunization of mucous membranes.	**[** **82** **-** **84** **, ** **102** **, ** **106** **, ** **117** **]**
**Gram-negative bacterial Lipopolysaccharide**	10-20 KDa	Safe if the mass ratio between the immunostimulatory agent and the antigen is above 500	Production of both IgG e IgA antibodies	**[** **85** **]**
**Ionic nanoparticles**	100-600 nm	Minimal or no toxicity	Excellent stability and facilitated delivery of the vaccine to the nasal mucosa	**[** **86** **]**
**Micellar nanoparticle**	6-100 nm	Minor side toxic effects than other adjuvants	Stability of the nanoparticles and facilitated antigen presentation	**[** **87** **]**
**Chitosan nanosphere**	10-1000 nm	No airway hyper-reactivity	Significant induction of IgG serum response and secretory IgA levels. INF-γ production in lung and spleen	**[** **96** **, ** **116** **]**
**Liposomes**	25-2500 nm	No toxicity up to 3000-4000 µM	Immune effect is similar to the intramuscular effect.	**[** **97** **]**
W805EC*	1-500 nm	All the components are on the FDA list of inactive ingredients for approved drugs	Enhanced uptake and delivery of the stabilized protein antigen to antigen presenting cells.	**[** **99** **-** **101** **]**
**Cross-linking oligo-chitosan and lymphotactin encoding plasmids**	250-350 nm	Safe pharmaceutical excipient for non-parenteral, non-blood contact	Systemic Th1-type immune response.	**[** **104** **]**
SPO1**	90-200 nm	High safety	Low cost, avoidance of cross- infection, suitable for people in different age groups	**[** **105** **]**
**Cationic nanogels**	1-1000 nm	Safe due to the non-deposition of the antigen in the olfactory bulb or central nervous system	Efficient adhesion to the nasal epithelium, effective and continuous delivery to the dendritic cells. Ability of function as an artificial chaperone.	**[** **107** **, ** **108** **]**
**Ion exchange or adsorbent resins**	< 2 x 10^5^ nm	Resins are inactive and harmless to the human body and contain no harmful impurities.	Both positive and negative charged antigens are easily transferred and released to the nasal mucosa.	**[** **113** **]**

*oil, purified water, nonionic detergent, organic solvent, and cationic surfactant. (60% W805EC = purified water, soybean oil, dehydrated alcohol, Polysorbate 80 NF, and cetylpyridinium chloride).

**squalene, polyoxyethylene, castor oil, and polyether.
